# Decomposition of factors associated with housing material inequality in under-five deaths in low and middle-income countries

**DOI:** 10.1186/s13690-021-00768-0

**Published:** 2022-01-04

**Authors:** Oyewale Mayowa Morakinyo, Adeniyi Francis Fagbamigbe, Ayo Stephen Adebowale

**Affiliations:** 1 Department of Environmental Health Sciences, Faculty of Public Health, College of Medicine, University of Ibadan, Ibadan, Nigeria; 2 Department of Epidemiology and Medical Statistics, Faculty of Public Health, College of Medicine, University of Ibadan, Ibadan, Nigeria

**Keywords:** Under-five deaths, Housing material inequality, Low-and middle-income countries

## Abstract

**Background:**

Low-and Medium-Income Countries (LMIC) continue to record a high burden of under-five deaths (U5D). There is a gap in knowledge of the factors contributing to housing materials inequalities in U5D. This study examined the contributions of the individual- and neighbourhood-level factors to housing materials inequalities in influencing U5D in LMIC.

**Methods:**

We pooled data from the most recent Demographic and Health Surveys for 56 LMIC conducted between 2010 and 2018. In all, we analysed the data of 798,796 children living in 59,791 neighbourhoods. The outcome variable was U5D among live births within 0 to 59 months of birth. The main determinate variable was housing material types, categorised as unimproved housing materials (UHM) and improved housing materials (IHM) while the individual-level and neighbourhood-level factors are the independent variables. Data were analysed using the Fairlie decomposition analysis at α = 0.05.

**Results:**

The overall U5D rate was 53 per 1000 children, 61 among children from houses built with UHM, and 41 among children from houses built with IHM (*p* < 0.001). This rate was higher among children from houses that were built with UHM in all countries except Malawi, Zambia, Lesotho, Gambia, Liberia, Sierra Leone, Indonesia, Maldives, Jordan, and Albania. None of these countries had significant pro-IHM inequality. The factors explaining housing inequalities in U5D include household wealth status, residence location, source of drinking water, media access, paternal employment, birth interval, and toilet type.

**Conclusions:**

There are variations in individual- and neighbourhood-level factors driving housing materials inequalities as it influences U5D in LMIC. Interventions focusing on reducing the burden of U5D in households built with UHM are urgently needed.

## Background

The implementation of the adopted international framework of the United Nations Convention on the Rights of the Child and other global interventions including the Global Strategy for Women’s, Children’s and Adolescent’s Health, the Millennium Development Goals, and the Sustainable Development Goals (SDGs) [1] by the global community, have led to a significant reduction in childhood deaths worldwide. Globally, there has been a decline in under-five deaths (U5D) from 93 deaths per 1000 live births in 1990 to 38 deaths per 1000 live births in 2019 [2]. A reduction in U5D by more than two-thirds were recorded in more than 80 countries inclusive of 31 Low- and lower-middle-income countries in 2019 (LMIC) [3].

**Fig. 1:**
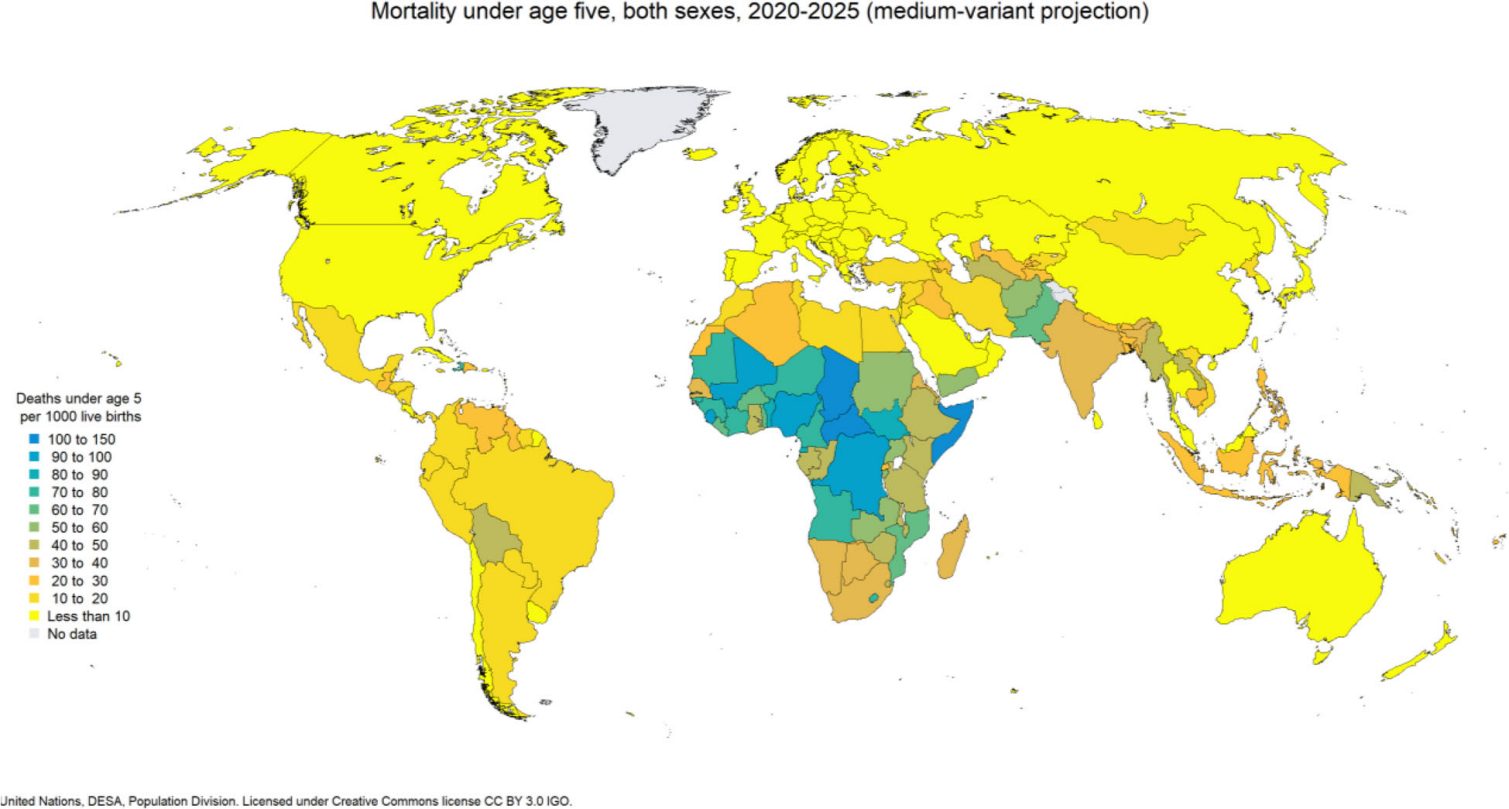
Distribution of under-five mortality rate worldwide

Despite these achieved levels of U5D reduction worldwide, U5D remains unacceptably high across certain countries and regions of the world (Fig. 1) [4]. Some of these countries are found in Africa, Asia and to some extent in Latin America. Countries with the highest U5D include the Democratic Republic of Congo, Ethiopia, India, Nigeria, and Pakistan [2, 3]. In 2019, 5.2 million children under age five died globally representing 14,000 deaths daily [5]. More than half of these deaths occurred in sub-Saharan Africa. The under-five mortality rate was 41 deaths per 1000 live births in LMIC as compared to 5 deaths per 1000 live births in high-income countries in 2019 [3].

Studies have shown that infectious diseases, including acute respiratory infections, measles, malaria and diarrhoea are leading direct causes of U5D, while the indirect causes are most especially the socio-economic, environmental and behavioural factors [6]. Difficulties associated with accessing fundamental lifesaving care such as the skill of birth attendant, postnatal care, adequate dietary intake, and vaccinations against common childhood diseases also predispose children to the risk of dying before the age of five years. Other factors that have been linked with U5D include the child gender, weight at birth, birth interval, and birth order, multiple births, maternal education, maternal age, marital status, maternal and paternal occupation, sex of the head of the household, access to media, sources of drinking water, toilet type, type of cooking fuel, household wealth index, place of residence and the type of housing materials with which a house is built [2, 7]. The well-being of household occupants is also a reflection of the building materials with which the house is built [8, 9].

Children’s homes are a key determinant of their environment [10]. It is also one of the major determining factors in their health outcome [11]. Poor housing conditions are one of the ways through which social and environmental inequalities lead to health inequality [11]. Poor housing conditions can elicit a range of diseases including respiratory infections, mental and behavioural dysfunction and neurological disorders which impact negatively on children’s health [12]. It is also a risk factor for U5D. A previous study has established the fact that the probability of a child dying before the age of 5 years was likely to be higher among children who lived in houses built with unimproved materials than those living in houses built with improved materials [9].

Literature is replete that inequality exists in the proportion of U5D and housing type [9]. However, there is a gap in the literature on what factors contributes to this inequality. Understanding these factors that contribute to housing materials inequalities in U5D can provide useful information needed to further reduce the burden of U5D in LMIC. This study is therefore designed to identify and quantify the contributions of the factors that determine the inequalities in U5D among children from houses built with improved housing materials and unimproved housing materials.

## Methods

### Study design and data

Data for this cross-sectional study were obtained from ICF, the primary owner and implementer of Demographic and Health Surveys (DHS) across countries worldwide. These surveys are nationally representative household surveys conducted in LMIC. This study used data from 56 recent DHS conducted between 2010 and 2018 and available in the public domain as of 15th of September 2020 when the data were harvested from dhsprogram.com. The DHS uses a multi-stage, clustered and stratified sampling design with households as the sampling frame using an individual country’s most recent census as a sampling frame. Typically, the upper stages include states or districts, or regions depending on country-specific administrative nomenclatures. The last stage of the multi-stage sampling is the selection of clusters from which households are selected. The clusters are referred to as the primary sampling units (PSUs) [13, 14]. All eligible women (aged 15 to 49 years) and men (aged 15–65 years) within each sampled household were interviewed. The surveys were not self-weighting. Therefore, sampling weights were calculated for all participants to account for unequal selection probabilities as well as for non-response. The application of the sampling weights ensures that survey findings represent the full target population. The DHS data includes household data, women’s data, birth recode data and children recode data and men’s data. However, the present study made use of the children recode data. Further details of the sampling methodologies are available at dhsprogram.com. In all, we pooled the data of 798,796 children, living in 59,791 neighbourhoods nested within 56 countries.

### Outcome variable

The outcome variable in this study is under-five death (U5D). Under-5 death is a death among live births within the first five years of life. It is defined as all deaths within 0 to 59 months of birth [13, 15]. To ensure the completeness and correctness of this variable, mothers were asked to name all their livebirths within five years preceding the date of the study. They were then asked if each of those children were alive or dead. The dates of death, the ages at death for the dead children and survival status were then used to determine U5D. Under-5 death was coded as a dichotomous variable: Alive or Died before the 5th birthday.

### Main determinant variable

The main determinate variable is the quality of the materials used to build houses where children under 5 years of age live. It was derived from the three sub-variables. These are the materials used for the house floor, wall and roof. During the survey, the enumerators personally observed these housing materials. The DHS provided a guideline for the classification of materials used for any of these three parts of the house as either improved or not. The classifications are (i) the improved floor materials are cement, ceramic tiles, vinyl asphalt strips, parquet and polished wood while the unimproved floor materials are earth, sand, dung, rudimentary, wood planks, palm, bamboo, and others; (ii) the improved wall materials include cement, stone with lime/cement, cement blocks and bricks while the unimproved wall materials are no wall, cane/palm/trunks, dirt, rudimentary, bamboo with mud, stone with mud, uncovered adobe, plywood, and others (iii) the improved roof materials are cement and roofing shingles while the unimproved materials consist of no roof, thatch/palm leaf, sod, rudimentary, rustic mat, palm/bamboo, wood planks, cardboard, wood, and others [15–18]. Each of the improved floor, wall and roof materials were scored “1” while unimproved materials were scored “0”. We then summed these scores. Houses with > = 2 scores out of the maximum obtainable 3 scores were classified as houses built with improved housing materials (IHM) while houses with < 2 scores were categorized as houses built with unimproved housing materials (UHM).

### Independent variables

The independent variables consist of individual-level and neighbourhood-level factors identified in the literature to be associated with childhood deaths.

#### Individual-level factors

The children characteristics, mothers’ characteristics and the households’ characteristics constitute the individual-level factors. The children characteristics are sex (male, female), weight at birth (average+, small and very small), birth interval (firstborn, < 36 months and > =36 months) and birth order (1, 2, 3 and 4+), a child is a twin (single, multiple (2+). The maternal characteristics: maternal education (none, primary or secondary plus), maternal age [15, 19–23], marital status (never, currently and formerly married), maternal and paternal employment status (working or not working), health insurance (yes /no). The household characteristics include the sex of the head of the household (male or female), access to media (at least one of radio, television, or newspaper), sources of drinking water (improved or unimproved), toilet type (improved or unimproved), cooking fuel (clean fuel or biomass), housing materials (improved or unimproved) and household wealth index (poorest, poorer, middle, richer and richest), place of residence (rural or urban).

#### Neighbourhood-level factors

Neighbourhood was operationalized as the clustering of children. The DHS uses “clusters” as the PSU. People of the same cluster are very likely to share similar contextual factors [13, 14]. We regard children as “neighbours” if they belong to the same cluster. In this study, we computed neighbourhood socioeconomic status (SES) as a neighbourhood-level from the proportion of mothers within the same clusters without education, belonging to a household in the two lowest wealth quintiles, has no media access and unemployed using the principal component factor method.

### Statistical analyses

The analytical approach for this study included descriptive statistics, bivariable analysis and multivariable decomposition analysis. Descriptive statistics to show the distribution of the children’s background characteristics as well as the distribution of U5D among the children from houses with IHM and UHM by countries and characteristics. The bivariable analysis was conducted using the Z-test to determine the equality of proportions of U5D among the children from houses with IHM and UHM within each country and region (Table 1). Charts were used for visualization. The spatial distribution of under-five deaths per 1000 livebirths among children in houses with improved and unimproved housing materials are shown in Fig. 1. The maps were built in Microsoft Projects 2020.

**Table 1:** Distribution of sample characteristics by countries, regions and prevalence of under-five deaths in LMIC by the quality of housing material, 2010–2018

Country	Survey Year	Sample	Number of Neighbourhoods	UHM (%)	Under-5 Deaths per 1000 livebirth		
					Overall	UHM	IHM
Overall		798,796	59,791	58.8	53	*61	41
Eastern Africa		107,839	6163	81.5	52	*54	45
Burundi	2011	13,134	554	90.3	59	*60	41
Comoros	2012	3129	250	57.0	42	44	40
Ethiopia	2016	10,508	635	97.6	55	56	32
Kenya	2014	20,464	1544	71.2	44	*46	39
Malawi	2016	17,159	845	82.4	49	49	49
Mozambique	2011	10,950	598	85.1	74	*76	62
Rwanda	2014	7735	487	92.3	38	38	38
Tanzania	2015	9736	575	79.5	53	54	48
Uganda	2016	15,024	675	72.3	50	51	47
Middle Africa		74,834	2851	78.1	70	*76	50
Angola	2016	14,177	615	60.7	51	*60	38
Cameroon	2018	9211	404	61.3	61	*65	55
Chad	2015	18,359	620	95.3	98	99	83
Congo	2012	8858	366	52.9	50	52	48
Congo DR	2014	18,455	529	88.6	75	*77	59
Gabon	2012	5774	317	95.0	53	53	46
Southern Africa		26,797	2447	51.1	50	51	49
Lesotho	2014	2896	370	50.1	69	64	74
Namibia	2013	4902	517	65.3	44	47	38
South Africa	2016	3397	643	33.1	36	*55	26
Zambia	2018	9733	531	63.5	49	46	53
Zimbabwe	2015	5869	386	31.3	57	63	54
Western Africa		145,850	6021	61.4	81	*91	64
Benin	2018	13,349	551	52.7	70	*78	61
Burkina Faso	2010	14,887	569	85.4	89	*95	54
Cote d’Ivoire	2013	7585	344	42.5	82	*93	75
Gambia	2013	7961	278	55.5	41	40	42
Ghana	2014	5757	416	48.0	47	48	46
Guinea	2018	7839	396	50.2	87	*106	68
Liberia	2013	7438	315	64.8	70	68	73
Mali	2018	9883	344	77.6	72	*82	36
Niger	2012	12,511	475	94.5	81	*83	48
Nigeria	2018	33,480	1370	51.0	97	*120	73
Senegal	2018	6492	208	19.8	39	*52	36
Sierra Leone	2013	11,842	432	69.5	114	111	119
Togo	2013	6826	323	57.0	62	*75	46
Central Asia		10,146	652	36.0	29	32	27
Kyrgyz Rep	2012	4135	300	23.7	26	33	25
Tajikistan	2017	6011	352	43.5	30	32	28
South-Eastern Asia		17,529	1834	62.9	26	*30	21
Cambodia	2014	7081	604	85.7	29	*32	10
Philippines	2017	10,448	1230	46.4	24	26	23
Southern Asia		322,711	31,390	48.7	44	*52	36
Afghanistan	2015	32,398	945	91.2	47	*48	35
Bangladesh	2014	7214	552	69.1	41	*45	30
India	2016	245,866	26,848	41.3	44	*55	37
Indonesia	2017	17,181	1894	54.6	27	25	28
Maldives	2016	3082	260	8.0	18	14	19
Nepal	2016	4674	352	62.8	33	35	30
Pakistan	2018	12,296	539	66.4	66	*74	51
Western Asia		27,617	1986	26.8	33	*45	28
Armenia	2016	1711	305	7.8	05	05	05
Jordan	2017	10,145	918	3.6	18	11	18
Yemen	2013	15,761	763	42.4	45	47	43
Central America		22,747	1948	60.4	28	*32	22
Guatemala	2014	12,252	845	50.8	31	*39	23
Honduras	2011	10,495	1103	72.4	25	27	21
South America		9022	1318	45.9	17	*21	14
Peru	2012	9022	1318	45.9	17	*21	14
Southern Europe		2722	647	2.4	4	3	4
Albania	2018	2722	647	2.4	4	3	4
Caribbean		21,755	1796	55.6	45	*50	39
Dominican Rep	2013	3549	491	11.4	28	*51	26
Haiti	2016	6378	438	45.7	67	73	62
Myanmar	2015	4684	419	87.2	44	*46	28
Timor Leste	2016	7144	448	67.1	37	*41	28
Oceania		9227	738	99.2	40	40	23
Papua NG	2016	9227	738	99.2	40	40	23
Total		798,796	59,791	58.8	53	*61	41

*significant at 5% test of equality of proportions

**Fig. 2:**
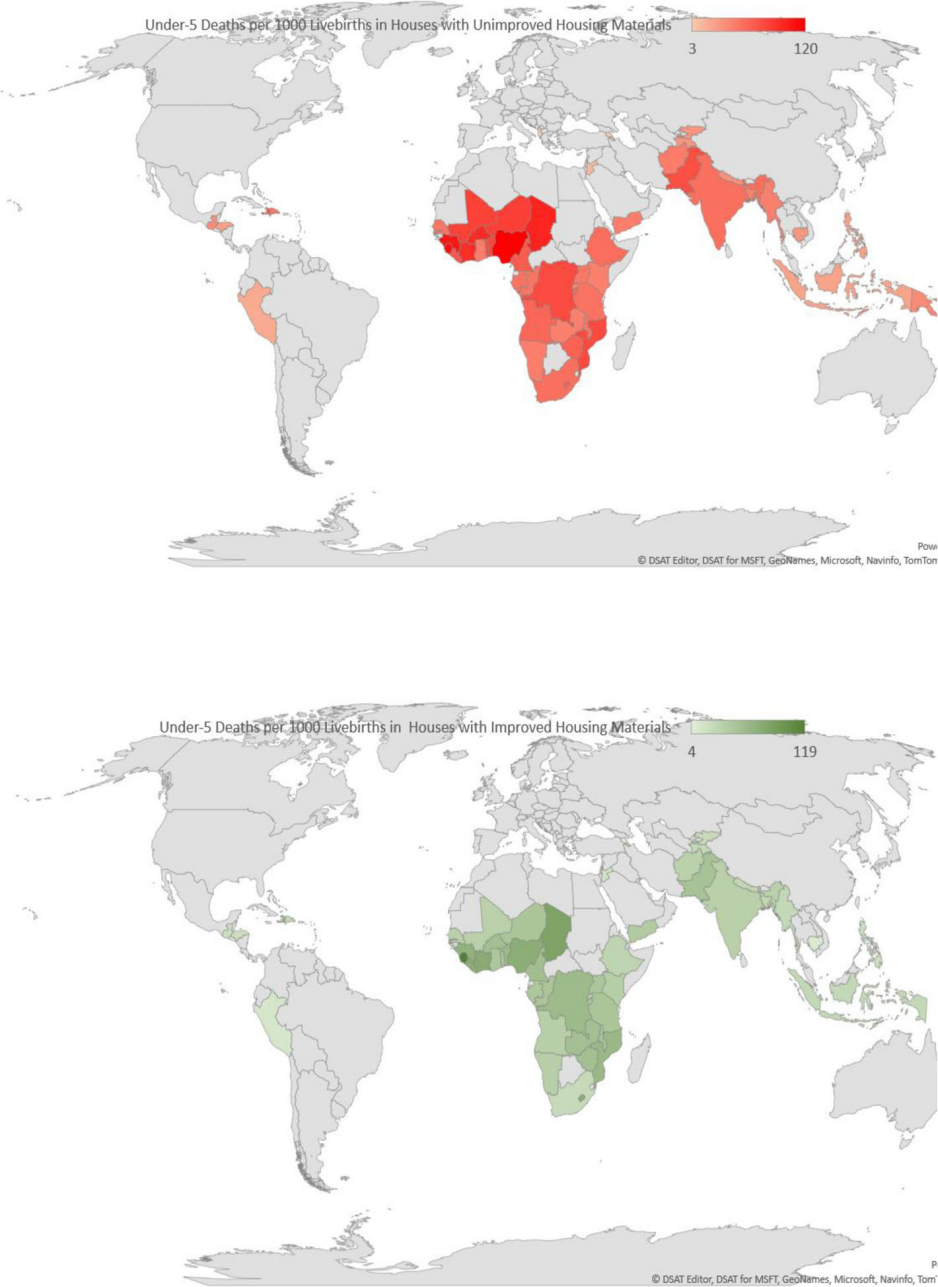
**a** Spatial distribution of under-five deaths among children in houses with unimproved housing materials in the LMIC studied. **2b** Spatial distribution of under-five deaths among children in houses with improved housing materials in the LMIC studied

We calculated the risk differences (RD) in U5D among the children from houses with IHM and UHM. A risk difference greater than 0 suggests that U5D are higher among the children from houses built with UHM than those from IHM (pro-unimproved housing material). Conversely, a negative RD indicates under-5 deaths are higher among the children from houses with IHM than those from UHM (pro-improved housing material). We carried out a country-level meta-analysis of the prevalence of U5D in each of the countries by computing the risk difference in the development of U5D between U5C from houses with improved and unimproved housing materials and presented the results in Fig. 2. A random-effects meta-analysis was used on the assumption that each country is estimating a study-specific true effect. We implemented the meta-analysis in R software by specifying the summary measure (SM) as risk difference (RD), the number of deaths in houses with improved and unimproved housing materials as well as the numbers of participants for each country, grouped by regions using the “metabin” command in R. We built a 95% confidence interval (CI) around the RDs to determine their significance.

The Mantel-Haenszel (MH) Odds Ratio (OR) and tests of heterogeneity of ORs were conducted to ascertain that the countries are different with regards to the odds ratio of U5D among children from houses with IHM and UHM and a test of homogeneity of ORs among all the countries with a significant odds ratio of U5D to determine if the odds of having U5D in those countries are homogenous. Finally, the Fairlie decomposition analysis (FDA) techniques using logistic models was applied.

Sampling weights were applied in all the analyses in this study to adjust for unequal cluster sizes, stratifications and to ensure that our findings adequately represent the target population. Multicollinearity among the independent variables was tested using the “colin” command in Stata version 16. The command provided the variance inflation factor (VIF). The VIF is approximate of the 1/(1-*R*^*2*^) ranging from 1 to infinity. The *R*^2^-value is obtained by regressing *tj*^*th*^ independent variable on other independent variables. All variables with VIF > 2.5 were removed from the regression analysis. Literature has shown concerns about VIF > 2.5 [24].

The FDA technique is an offshoot of the well-known Blinder-Oaxaca decomposition analysis technique that was originally developed for linear models [25–27]. FDA was developed following the inefficiency of the Blinder-Oaxaca decomposition analysis technique in handling non-linear outcomes such as logit or probit models [19, 20, 28–30]. The FDA was developed for non-linear regression models and used in the quantification of the contributions to differences in the prediction of an outcome of interest between two groups [31]. This technique is a counterfactual method with an assumption that “what the probability of under-5 death would be if children from houses built with UHM had the same characteristics as the children whose houses are built with IHM?”

The FDA allows for the decomposition of the difference in an outcome variable between 2 groups (children from houses with IHM and UHM) into 2 components. The first component is the “explained” (also referred to as the “compositional” or “endowments”) portion of that gap that captures differences in the distributions of the measurable characteristics. The explained part is the portion of the gap in U5D attributable to the differences in observable, measurable characteristics between children from houses with IHM and UHM. This method helps to quantify how much of the gap between the children from houses with UHM and the children from houses with IHM is attributable to these differences in specific measurable characteristics. The second component of the model is the “unexplained” (also referred to as the “structural” component or the “coefficient”) part. The unexplained part is the portion of the gap due to the differences in the estimated regression coefficients and the unmeasured variables between the two groups.

The Fairlie decomposition technique works by constraining the predicted probability between 0 and 1 as available in a logit model. The coefficients (*β*) estimated by the logit regression technique with the probability of under-5 deaths conditioned on the independent variables (*X*) is obtained as

$$ \mathit{\Pr}\left(U5D=1|X\right)=\frac{e^{X^{\prime}\beta }}{1+{e}^{X^{\prime}\beta }}\dots \dots \dots \dots \dots \dots .(1) $$

We carried out an FDA analysis by calculating the difference between the predicted probability for Group A (children from houses with UHM) using the Group B (children from houses with UHM) regression coefficients and the predicted probability for under-5 deaths among Group B using its regression coefficients [19].

Fairlie et al. showed that the decomposition for a nonlinear equation *Y* = *F*(*X*), can be expressed as:

$$ {\overline{\mathrm{Y}}}^A-{\overline{\mathrm{Y}}}^B=\overset{1^{st}}{\overbrace{\left[\sum \limits_{i=1}^{N^A}\frac{F\left({X}_i^A{\hat{\beta}}^A\right)}{N^A}-\sum \limits_{i=1}^{N^B}\frac{F\left({X}_i^B{\hat{\beta}}^A\right)}{N^B}\right]}}+\overset{2^{nd}\ }{\overbrace{\left[\sum \limits_{i=1}^{N^B}\frac{F\left({X}_i^B{\hat{\beta}}^A\right)}{N^B}-\sum \limits_{i=1}^{N^B}\frac{F\left({X}_i^B{\hat{\beta}}^B\right)}{N^B}\right]}}\dots \dots \dots (2) $$

Where *N*^*A*^ is the sample size for group *J* [32]. In equation (1), $\overline{\mathrm{Y}}$ is not necessarily the same as $F\left(\overline{\mathrm{X}}\ \hat{\beta}\right)$, unlike in BODA where *F*(*X*_*i*_*β*) = *X*_*i*_*β*. The 1st term (explained) is the part of the gap in the binary outcome variable that is due to group differences in distributions of *X*, and the 2nd term (unexplained) is the part due to differences in the group processes determining levels of *Y* (under-5 deaths). The 2nd term also captures the portion of the binary outcome variable gap due to group differences in unmeasurable or unobserved endowments.

The estimation of the total contribution is the difference between the average values of the predicted probabilities. Using coefficient estimates from a logit regression model for a pooled sample, ${\hat{\beta}}^{\ast }$, the independent contribution of *X*_1_ and *X*_2_ to the group, the gap can be written as

$$ \frac{1}{N^B}\times \sum \limits_{i=1}^{N^B}F\left({\hat{\alpha}}^{\ast }+{X}_{1i}^A{\hat{\beta}}_1^{\ast }+{X}_{2i}^A{\hat{\beta}}_2^{\ast}\right)-F\left({\hat{\alpha}}^{\ast }+{X}_{1i}^B{\hat{\beta}}_1^{\ast }+{X}_{2i}^A{\hat{\beta}}_2^{\ast}\right)\dots \dots \dots (3) $$

and

$$ \frac{1}{N^B}\times \sum \limits_{i=1}^{N^B}F\left({\hat{\alpha}}^{\ast }+{X}_{1i}^B{\hat{\beta}}_1^{\ast }+{X}_{2i}^A{\hat{\beta}}_2^{\ast}\right)-F\left({\hat{\alpha}}^{\ast }+{X}_{1i}^B{\hat{\beta}}_1^{\ast }+{X}_{2i}^B{\hat{\beta}}_2^{\ast}\right)\dots \dots \dots (4) $$

respectively. The contribution of each variable to the gap is thus equal to the change in the average predicted probability from replacing the group *B* distribution with the group *A* distribution of that variable while holding other variables constant. Other detailed numerical of this approach have been reported in the literature [19, 20, 30, 32, 33]. We implemented the FDA in STATA 16 (StataCorp, College Station, Texas, United States of America) using the “Fairlie” command.

## Results

Table 1 shows the distribution of U5D across children from houses built with UHM and IHM by countries and regions. The overall prevalence of houses with UHM was 58.8%. The highest proportion of children from houses built with UHM was found in Papua New Guinea (99.2%) and least in Albania at 2.4%. The overall weighted prevalence of U5D was 53 per 1000 children, 61 among children from houses built with UHM and 41 among children from houses built with IHM (*p* < 0.001). The prevalence of U5D among children from houses built with UHM ranged from 3 per 1000 children in Albania to 120 in Nigeria, while it ranged from 4 in Albania to 119 in Sierra Leone among children from houses built with IHM. The spatial distribution of U5D per 1000 livebirths among children in houses with improved housing materials are shown in Fig. 2(a) and (b), respectively.

Table 2 shows the descriptive statistics of the characteristics of the children across the 56 LMIC and prevalence of U5D by the quality of housing materials. The overall U5D was highest among multiple births from houses built with UHM (23.3%) compared with 15.8% from houses built with IHM. Also, the prevalence of U5D was highest among females than males (59.2% vs 58.5%), among children using unimproved water sources (80.8%) and unimproved toilets (77.1%).

**Table 2:** Summary of pooled background characteristics of the studied children and prevalence of under-five deaths in LMIC by the quality of housing material, 2010–2018

Characteristics	sample	%	UHM (%)	Under-5 Deaths per 1000 livebirth		
				Overall	UHM	IHM
Maternal Current Age						
15–24	233,276	29.2	59.7	54	62	43
25–34	413,113	51.7	56.2	48	57	37
35–49	152,407	19.1	64.5	62	69	49
Maternal Highest Education		0.0				
No Education	283,668	35.5	75.8	70	73	59
Primary	207,148	25.9	70.1	55	57	50
Secondary+	307,980	38.6	36.4	36	43	32
Media		0.0				
No	332,556	41.7	78.2	66	68	59
Yes	465,573	58.3	45.8	44	53	36
Maternal Employment		0.0				
Employed	306,061	54.0	67.5	63	68	51
Unemployed	260,867	46.0	61.3	46	53	35
Paternal Employment		0.0				
Employed	512,803	95.7	64.9	56	63	43
Unemployed	22,993	4.3	65.1	48	53	40
Marital status		0.0				
Never married	25,286	3.2	55.7	54	57	49
Currently married	738,686	92.5	58.6	52	61	40
Formerly	34,822	4.4	65.2	66	70	58
Sex of household head		0.0				
Male	670,970	84.0	58.9	53	61	41
Female	127,826	16.0	58.1	52	58	42
Wealth index combined		0.0				
Poorest	206,972	25.9	89.8	64	65	49
Poorer	180,011	22.5	74	59	63	49
Middle	158,224	19.8	54.6	52	58	45
Richer	138,947	17.4	38.2	46	55	41
Richest	114,642	14.4	21.6	36	46	33
Covered by health insurance		0.0				
No	635,801	87.8	60.4	56	64	44
Yes	88,596	12.2	41.3	34	41	29
Child is twin		0.0				
Single birth	777,993	97.4	58.8	49	56	38
Multiple	20,803	2.6	60.7	203	233	158
Sex of child		0.0				
Female	389,073	48.7	59.2	49	56	38
Male	409,723	51.3	58.5	56	65	44
Weight at birth		0.0				
Average+	633,173	84.0	57.7	45	54	34
Small	87,302	11.6	62.4	68	76	53
Very small	33,169	4.4	66.8	116	113	123
Birth order						
1	219,586	27.5	49.9	50	62	38
2	189,166	23.7	51.5	42	50	33
3	129,778	16.2	59.2	48	53	40
4+	260,266	32.6	72.2	66	70	58
Birth interval						
1st Birth	219,591	27.6	49.9	50	62	38
< 36 months	316,485	39.7	65.4	65	73	49
36+ months	260,722	32.7	58.8	39	42	34
Drinking water						
Unimproved sources	186,576	23.4	80.8	67	70	54
Improved source	612,220	76.6	52.8	49	57	40
Toilet type						
Unimproved sources	410,192	51.4	77.1	63	66	54
Improved source	388,378	48.6	40.2	42	51	36
Cooking fuel						
Unclean/biomass	617,796	77.4	70.4	60	64	52
Clean fuel	180,324	22.6	24.6	30	36	28
Place Of Residence						
Urban	235,866	29.5	32.5	42	52	37
Rural	562,930	70.5	70.9	58	63	46
Community SES Disadvantage						
Least	160,002	20.1	27.7	34	43	30
2	159,515	20.0	43.6	47	53	42
3	159,642	20.0	71.1	57	61	48
4	159,251	20.0	74.5	63	67	49
Highest	158,401	19.9	78.8	63	67	53
Total	796,672	100.0	58.8	53	61	41

### Risk differences in U5D among children from UHM and IHM

**Fig. 3:**
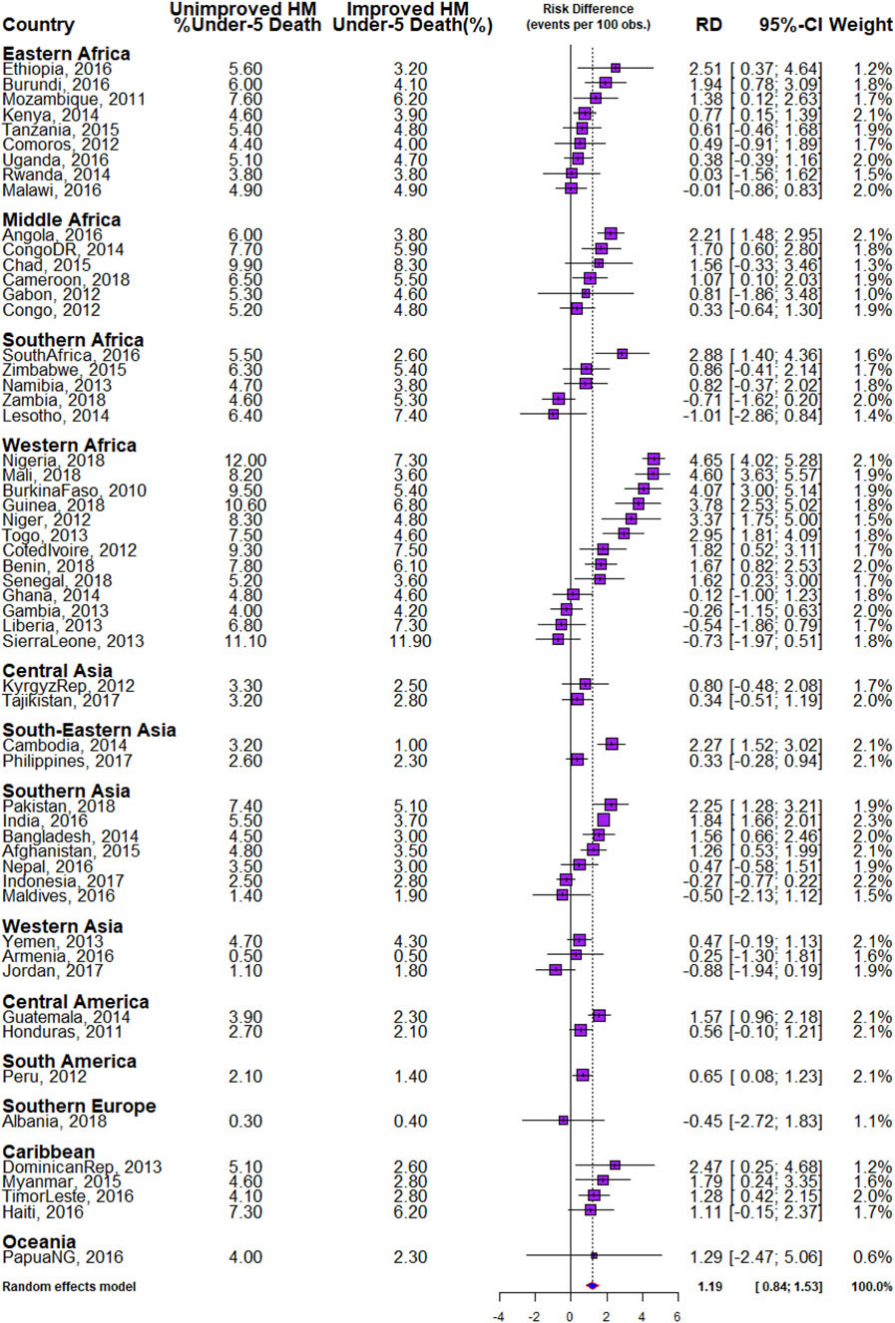
Forest plot of the risk difference in the prevalence of under-five deaths by the improvement of housing materials in LMIC

**Fig. 4:**
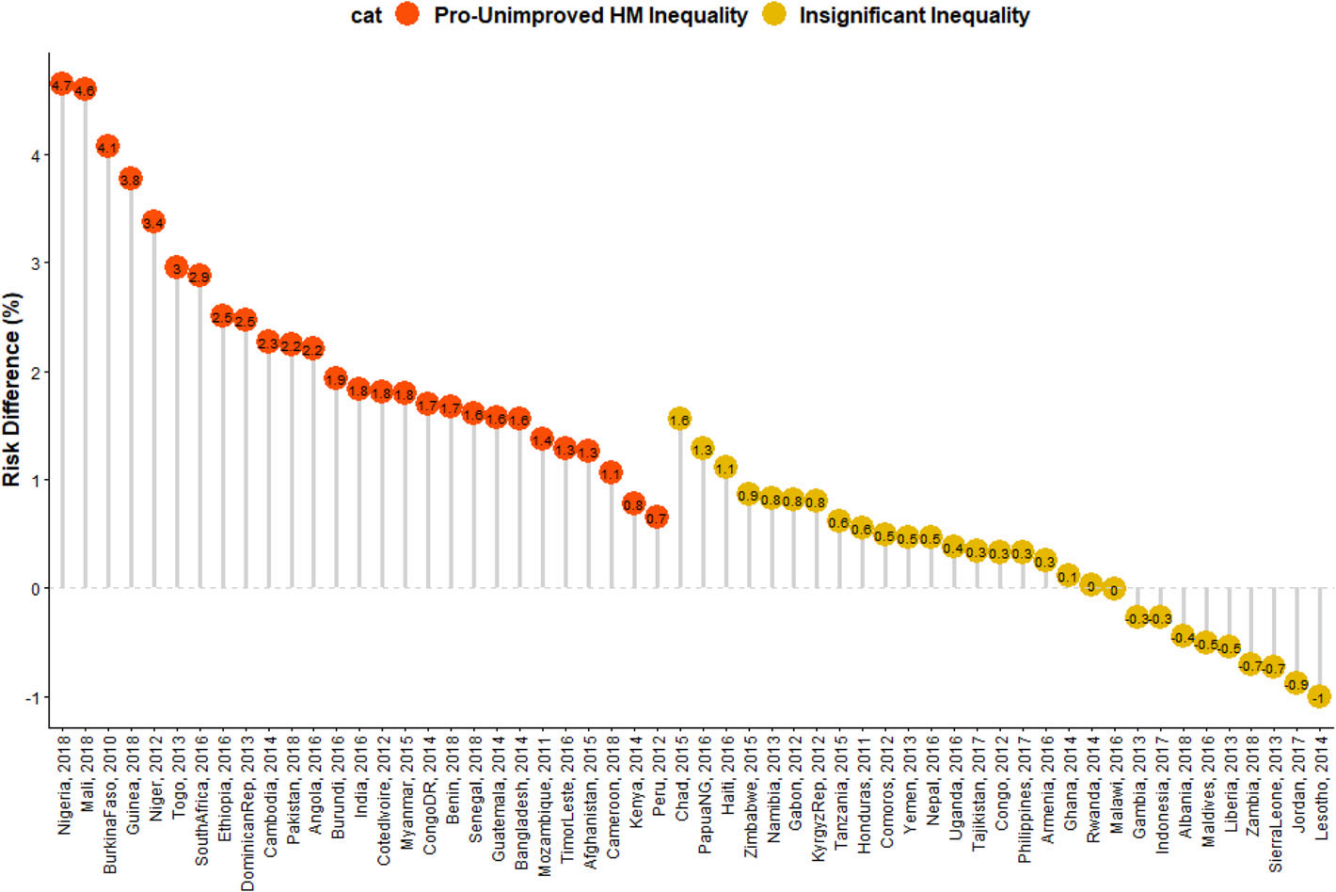
Risk difference between children from houses with improved and unimproved housing materials in the prevalence of under-five deaths by countries in LMIC

**Fig. 5:**
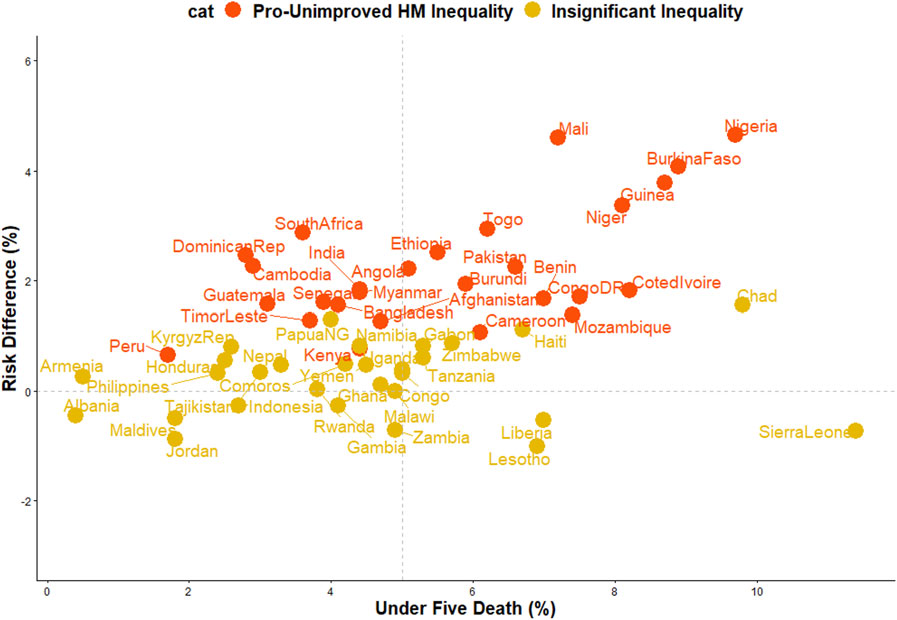
Scatter plot of rate of under-five deaths and risk difference between children from houses with improved and unimproved housing materials in LMIC

The risk differences of U5D among children from houses built with UHM and IHM across the countries studied are presented in Figs. 3, 4 and 5 The prevalence of U5D was generally higher (RD > 0) among children from houses built with UHM in all the countries (pro-UHM inequality) except in Malawi, Zambia, Lesotho, Gambia, Liberia, Sierra Leone, Indonesia, Maldives, Jordan and Albania with RD < 0. None of these countries had significant pro-IHM inequality. The heterogeneity of the RDs was 88% (*p* < 0.01).

Irrespective of regions, the fixed effects of pro-UHM differences in U5D were largest in Nigeria (46.5/1000 children) while the fixed effects of pro-IHM RD were largest for Lesotho (− 9.9/1000). The random effect, which is the RD of U5D irrespective of country of residence was 11.8/1000 children (95% CI: 8.4–15.3). The greatest contribution to the weight of the random effect was found in India at 2.3% while the least was in Papua New Guinea at 0.6% as shown in Fig. 3.

### Risk difference and prevalence of under-5 deaths and magnitude of housing material inequality

In Figs. 4 and 5, red and orange colours were used to depict the countries with significant pro-UHM inequality and insignificant inequality respectively. There was no statistically significant pro-IHM inequality in any of the countries. Twenty-four countries showed a statistically significant pro-UHM inequality (Figs. 3, 4 and 5).

### Relationship between the prevalence of U5D and magnitude of inequality

The relationships between the prevalence of U5D and the magnitude of the inequality in the houses (whether built with improved or unimproved materials) where the children’s lives are presented in Fig. 4. Countries with high U5D and high pro-UHM inequality include Cote D’Ivoire, Nigeria, Mali, Niger and Burkina Faso while countries with high U5D and high pro-IHM inequality include Sierra Leone, Liberia and Lesotho. Dominican Republic, India, Cambodia, and South Africa are examples of countries with low U5D and high pro-UHM inequality while Jordan and Gambia are low U5D and high pro-IHM inequality countries.

### Decomposition of factors in the prevalence of U5D by inequalities in the improvement of housing materials

Of the 56 countries, statistically significant pro-UHM was found in only 26 countries. The countries are Afghanistan, Angola, Bangladesh, Benin, Burkina Faso, Burundi, Cambodia, Cameroon, Congo DR, Cote d’Ivoire, Dominican Rep, Ethiopia, Guatemala, Guinea, India, Kenya, Mali, Mozambique, Niger, Nigeria, Pakistan, Peru, Senegal, South Africa, Timor Leste, and Togo.

The test of homogeneity of the odds of U5D among the children from the pro-UHM countries showed that ORs: *X*^*2*^ = 95.10, d.f. = 25, and *p* = 0.001. The MH-OR of having U5D among children in the 26 countries with pro-UHM inequalities was 1.42 (95% CI: 1.38–1.46).

**Fig. 6:**
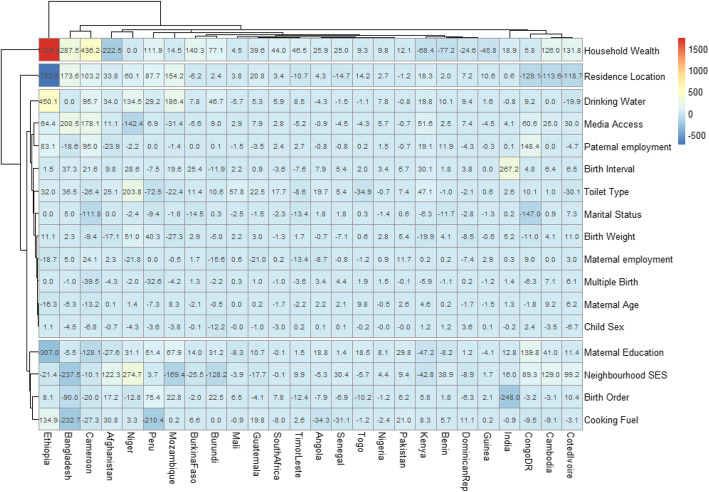
Contributions of differences in the distribution ‘compositional effect’ of the determinants of under-five deaths to the total gap between children from houses with improved and unimproved housing materials among countries with pro-rural inequality

Figure 6 shows the detailed decomposition of the part of the pro-UHM inequality caused by compositional and structural effects of the factors associated with U5D in the 26 LMIC with pro-U5H inequality. The red boxes in the heat map are the “explained” (compositional component) while the “unexplained” (structural component) portions of the pro-UHM inequalities are depicted in blue boxes as shown in Fig. 5. The lighter the red colour, the lower the percentage contribution of the “explained” portion and the lighter the blue colour, the lower the percentage contribution of the “unexplained” portion.

We found wide variations in the factors associated with the pro-U5H inequalities across the countries. There was clustering among household wealth status, residence location, source of drinking water, media access, paternal employment, birth interval and toilet type while maternal education, neighbourhood SES, birth order and cooking fuel formed another cluster. All these factors contributed most to the inequalities. Also, Ethiopia, Bangladesh, Cameroun, Afghanistan and Niger formed a cluster of countries with similar associated factors with U5D while India, Congo DR, Cambodia and Cote d’Ivoire formed another cluster.

Different factors had the largest association with U5D in different countries. In Ethiopia, the greatest contributions to pro-UHM equalities are household wealth status (1728% - 17 times higher odds of death among children from the richest household compared with those from poorest households), residence location (752%), drinking water sources (450%), maternal education (307%) and cooking fuel (135%). Neighbourhood SES (236%), household wealth status (288%), cooking fuel (233%), media access (201%) and residence location (174%) contributed most to pro-UHM in Bangladesh. In Cote d’Ivoire, the largest contributions to pro-UHM inequality in U5D were household wealth status (132%), residence location (119%), and neighbourhood SES (99%).

## Discussions

This study identified factors that contributed to housing material inequality in U5D in 56 LMIC, on which limited research has previously been conducted. The most salient findings show that [1] a higher prevalence of U5D was recorded among children living in houses built with UHM in 46 out of 56 countries studied [2] there was no statistically significant pro-IHM inequality in any of the countries but significant pro-UHM was recorded in only 26 countries [3] factors associated with the pro-UHM and pro-IHM inequalities in U5D differ widely across countries.

The highest U5D was recorded among twin births compared with singletons. The rates were 23.3% vs 5.6% among children from houses built with UHM and 15.8% vs 3.8% among children from houses built with IHM. A startling one-fifth of twins died before attaining age 5 years in sub-Saharan Africa. The risk of dying is three times higher among multiple births compared to singletons [34]. The rate of U5D is two to five times higher among twin birth compared to singletons in regions known for low childhood deaths [34] and in sub-Saharan countries where the highest global U5D has been observed in recent times [35, 36]. There is a higher probability of U5D among twins than singletons in households sourcing water from unimproved sources. Since twins start complimentary food much earlier than singletons, the use of unimproved water in the preparation of complementary foods could contaminate such foods with gastroenteritis which are risk factors for communicable diseases and malnutrition [21, 22]. This may explain the higher U5D recorded among twin births. Our study showed evidence of higher U5D among children from households with unimproved water sources and unimproved toilets. Drinking water from unimproved sources and the use of unimproved toilets make children more vulnerable to diarrhoea, parasitic and helminthic infections which may compromise their nutritional status and make them susceptible to poor health outcomes [37–39].

Moreover, the proportion of pro-UHM inequality (99.2%) was highest in Papua New Guinea (PNG) and least in Albania (2.4%). Papua New Guinea is a low resource, and socioeconomically diverse country [40, 41] with the majority of the people occupying the low socio-economic cadre and thus cannot access quality and affordable housing [42, 43]. Although PNG has the highest number of houses built with UHM, it did not translate to high U5D in the country. This might be unconnected with the non-existence of reliable data to measure childhood mortality in PNG. PNG relies mostly on the use of civil registration and routine health data which have been reported to be incomplete, deficient and inaccurate for measuring childhood mortality in the country [44].

Findings from this study show that Nigeria has the highest rate of U5D among children from houses with UHM. This result gave credence to the study by Adebowale et al. who investigated if housing materials were a predictor of U5D in Nigeria using the DHS 2013 dataset [9]. They reported that the hazard of U5D was 1.46 higher among children who lived in houses with UHM than those in IHM. The refined U5D estimates in houses with UHM and IHM was 143.5 and 90.8 per 1000 live births, respectively [9]. Living in houses built with unimproved materials can predispose children to a range of health morbidities that culminate in deaths. For instance, the odds of malaria infection was significantly higher among under-five children who lived in houses built completely with unimproved materials than those living in improved housing materials in Nigeria [16]. In 2018, children under-5 years made up 67% (272000) of global malaria deaths. Nigeria accounted for 24% of all malaria global deaths [45]. In another study conducted among under-five children in Nigeria, more cases (65.2%) than controls (42.4%) who were diagnosed with respiratory infections lived in houses of poor quality [46]. Pneumonia, a form of acute respiratory infection, is one of the highest causes of U5D in Nigeria killing more than 140,000 annually [47].

The RD in U5D between houses built with UHM and those built with IHM was obvious across countries. Forty-six countries recorded higher U5D (RD > 0) in houses with pro-UHM inequality than the ten countries with pro-IHM inequality (RD < 0). In the regions, statistically significant pro-UHM inequality in U5D was found in four Eastern countries (Burundi, Ethiopia, Kenya, Mozambique), nine Western African countries (Benin, Burkina Faso, Cote D’Ivoire, Guinea, Mali, Niger, Nigeria, Senegal, Togo), four Southern Asian counties (Afghanistan, Bangladesh, India, Indonesia), three in Caribbean countries (Dominican, Myanmar, Timor-Leste). Countries in South-Eastern Asia (Cambodia, Philippines), Oceania (Papua New Guinea), Central Asia (Kyrgyz Rep, Tajikistan), Western Asia (Armenia, Yemen, Jordan), Central America (Honduras), and South America (Peru) had no statistically significant pro-UHM inequality. An RD of 12 per 1000 children observed among countries with significant pro-UHM inequality suggests that more deaths will be recorded among children whose mothers lived in houses with UHM than those from houses with IHM. One probable reason for the differences in the housing materials inequalities can be attributed to country-level factors which cut across individual, household and neighbourhood factors and varies across LMIC [23, 48, 49]. There is the need for urgent intervention in LMIC by the government and relevant stakeholders through the provision of low-cost and sustainable housing, and the enforcement or initiation of policies and programmes that encourage the maintenance of existing housing units.

Moreover, our findings show the relationship between the prevalence of U5D and the magnitude of housing material inequality. Countries such as Maldives, Armenia, Jordan and Albania have U5D of less than 25 per 1000 births which were what the SDG is targeting. The WHO had reported an 80% reduction in the prevalence of U5D in Eastern and South-Eastern Asia countries [50]. This achievement was attributed to the political will of the government of these countries to implement primary health care and universal health coverage through improved access to quality care, free health services for mothers and children, family planning support and capacity building of health workers [50].

However, countries such as Nigeria, Mali, Niger, Burkina Faso and Guinea have high U5D and high-risk differences between children living in houses from UHM and IHM. A weak health system, political and economic instability among other factors that prevent mothers and their children from accessing quality lifesaving healthcare services have been identified as the cause of high U5D in Cote D’Ivoire, Nigeria, Mali, Niger and Burkina Faso. Countries such as Nigeria, Mali, Niger, Burkina Faso and Guinea should learn from other countries (Maldives, Armenia, Jordan and Albania) with low U5D and low-risk differences between living in houses from UHM and IHM, by taking urgent steps towards the realization of target 3.2 of the SDGs. It is interesting to note that there was no statistically significant pro-IHM inequality in any of the countries. This further shows the link between good housing quality and improved health and wellbeing of its inhabitants [8, 9, 51].

We found wide variations in the factors explaining the housing material differentials in the prevalence of U5D in LMIC. Children from households with poor wealth quintile, who resides in rural areas, drink water from unimproved sources, uses unimproved toilet types, have poor access to media, have short birth interval and fathers that are unemployed have a higher probability of having pro-UHM inequality in experiencing U5D. These findings are consistent with previous studies [7, 52, 53].

An individual choice of housing type, features, size and quality are dependent on several factors including socio-economic factors [54]. In LMIC, households with poor wealth quintile have limited choices and are more likely to live in houses built with inferior materials, and have less to spend on maintenance and repairs [54, 55] which invariably increases the likelihood of child death. The outcome of lengthy non-maintenance of buildings includes dilapidated structures, leaking ceilings and pipes, peeling off of paints and cracks and holes in walls and floors. Cracks on walls and floors provide a conducive environment for mites, respiratory viruses and cockroaches to breed; all of which elicit respiratory morbidity [9, 51, 56]. Also, housing disrepair can act as stressors thus compromising the human immune system [54].

Moreover, poverty is also a predictor of unclean fuel use in LMIC [57, 58]. When unclean fuel is burnt, it releases harmful pollutants such as respirable particulate matter and carbon monoxide into the atmosphere [59]. A child’s exposure to fumes from unclean fuel can suppress the functioning of the immune system while increasing bronchial reactivity, which promotes susceptibility to bacterial and viral pathogens [60, 61]. Respiratory infections from unclean fuel is a major cause of death among children [59, 62].

Nonetheless, the use of unimproved toilet facilities hinders the safer disposal of faeces and promotes the risk of contact between diarrhoea causative organisms and human hosts (Aziz et al., 2018). Diarrhoea is a major cause of U5D in LMICs [63–65]. Conversely, having an educated mother, living in a neighbourhood of high SES, high birth order and cooking with clean fuel reduces the probability of U5D and influenced the housing material inequalities in U5D across LMIC. This position has been reported in the literature [66, 67].

We found wide variations across countries in the factors associated with the pro-under five housing inequalities. The highest contributors to pro-UHM equalities in Ethiopia are household wealth status, residence location, drinking water sources, maternal education and cooking fuel. Neighbourhood SES, household wealth status, cooking fuel, media access and residence location contributed most to pro-UHM in Bangladesh while in Cote d’Ivoire, the largest contributions to pro-UHM inequality in U5D were household wealth status, residence location, and neighbourhood SES. The findings from our study bring to the fore the importance of enhancing the compositional and structural factors if housing materials inequalities in U5D are to be reduced. Integrated geographically specific interventions may be a better approach to tackling the housing materials inequalities in U5D in LMIC with policies and programmes tailored to country-specific needs.

### Policy implication

Housing is essential for human survival. Despite this, many people around the world still lack access to safe, appropriate, and affordable housing. Housing conditions have a significant impact on both individual and community health. This nexus is recognised by international human rights legislation, which establishes basic standards—standards that governments are legally bound to respect, preserve, and fulfil—that, if realised, will result in healthier living conditions for everyone, everywhere. Under international human rights legislation, particularly the Universal Declaration of Human Rights, the right to sufficient housing is firmly established and recognised.

Moreover, the Sustainable Development Goals (SDGs) were adopted by the United Nations in 2015 to preserve healthy lives and guarantee the well-being of all people. The goal 11 target 11.1 of the SDGs is to ensure access for all to adequate, safe and affordable housing and basic services and upgrade slums’ by the year 2030 [68]. Adequate, secure, and affordable housing provides an entry point to a more sustainable city.

However, despite the adoption of the SDGs by the government of most nations and the central place of housing in reducing preventable diseases and deaths among under-five children, the prevalence of USD associated with living in housing with unimproved materials is still unacceptably high. Findings from this study have clearly shown that inequalities still exist in housing types across most LMIC and that living in unimproved housing is a contributory factor to U5D.

To realise goal 3 target 3.2 of the SDGS, which is to end preventable deaths of newborns and children under 5 years of age by year 2030 and a reduction in U5D in LMIC, nations must take seriously their international legal commitments to respect, safeguard, and implement the right to appropriate housing. The rights enshrined in these agreements must be implemented and enforced. Also, the government and other key stakeholders should develop guidelines on healthy housing to help prevent a wide range of preventable illnesses that are frequently linked to poor housing conditions. The outcomes of such advocacy and actions would go a long way toward improving the health of the populace including children less than 5 years in age.

### Study strengths and limitations

This study is one of the first to demystify the factors associated with housing materials inequality in LMIC. The representative nature of data from 59 DHS, their large sample size and high response rate makes the quality of findings from the study useful for international comparison. The causal relationship between housing materials and U5D could not be determined because of the cross-sectional nature of this study. Also, the measure of child mortality using information obtained from mothers may underestimate the actual rate as a result of recall bias. The Fairlie decomposition analysis used in this study has some advantages over the Blinder-Oaxaca decomposition analysis technique in handling non-linear outcomes such as logit or probit model. It can effectively handle non-linear regression models and quantification of the contributions to an outcome of interest between two groups.

## Conclusions

In this study, we identified a high prevalence of U5D with significant pro-unimproved housing materials inequalities in most LMIC. This showed that the burden of U5D, which is disproportionately higher among children living in houses built with unimproved housing materials compared to those in houses built with improved materials was explained by the individual, household and community-level factors. The decomposition analysis revealed that factors such as wealth quintile, place of residence, drinking water sources, toilet types, media access, birth interval and paternal employment are the major contributors to housing inequalities in most LMIC. These findings can serve as a spur for planning children’s country-specific survival programs. Interventions targeted towards the use of good quality materials for the construction of houses and their subsequent maintenance can help reduce the burden of U5D and other related health morbidities, and thus, ensures the wellbeing of their inhabitants. Improving equity, economic productivity, and environmental sustainability would requires addressing the issue of adequate, secure, and affordable housing within and around the city. This will lead to a higher quality of life and better equality of opportunity, resulting in a more dynamic and just community.

## Data Availability

The datasets generated and/or analysed during the current study are available in the DHS repository, http://dhsprogram.com with Accession number 140625.

## Abbreviations

CI: Confidence Interval
DHS: Demographic and health survey
FDA: Fairlie decomposition analysis
IRB: Institutional review board
LMIC: Low- and middle-income countries
PSU: Primary sample unit
RD: Risk difference
SES: Socioeconomic status
PNG: Papua New Guinea
U5C: Under-five children
U5D: Under-five deaths
Under-5 Deaths: Under-five deaths
UNICEF: United Nations International Children’s Emergency Fund
WHO: World Health Organization
